# Postradiation Fractures after Combined Modality Treatment in Extremity Soft Tissue Sarcomas

**DOI:** 10.1155/2021/8877567

**Published:** 2021-03-15

**Authors:** Meredith K. Bartelstein, Divya Yerramilli, Alexander B. Christ, Shachar Kenan, Koichi Ogura, Tomohiro Fujiwara, Nicola Fabbri, John H. Healey

**Affiliations:** ^1^Department of Surgery, Orthopedic Service, Memorial Sloan Kettering Cancer Center, 1275 York Ave., New York, NY 10065, USA; ^2^Department of Radiation Oncology, Memorial Sloan Kettering Cancer Center, 1275 York Ave., New York, NY 10065, USA

## Abstract

Soft tissue sarcoma (STS) of the extremities is typically treated with limb-sparing surgery and radiation therapy; with this treatment approach, high local control rates can be achieved. However, postradiation bone fractures, fractures occurring in the prior radiation field with minimal or no trauma, are a serious late complication that occurs in 2–22% of patients who receive surgery and radiation for STS. Multiple risk factors for sustaining a postradiation fracture exist, including high radiation dose, female sex, periosteal stripping, older age, femur location, and chemotherapy administration. The treatment of these pathological fractures can be difficult, with complications including delayed union, nonunion, and infection posing particular challenges. Here, we review the mechanisms, risk factors, and treatment challenges associated with postradiation fractures in STS patients.

## 1. Introduction

The treatment of high-grade soft tissue sarcoma (STS) of the extremities traditionally involves a combination of surgery and radiation therapy (RT). The use of RT in either the neoadjuvant or the adjuvant setting significantly improves local control rates and allows for limb preservation in most cases [1, 2]. RT is therefore a critical component of treatment for these tumors. However, RT is associated with several serious skeletal complications, including radiation osteitis [3], avascular necrosis [4], stress fractures [5], delayed wound healing [6], growth plate arrest in skeletally immature patients [7], and fractures.

Postradiation fractures, those that occur in a prior radiation field and are associated with minimal or no trauma, pose a significant challenge for both patients and orthopedic surgeons. These fractures occur in 2–22% of STS patients treated with a combination of surgery and RT, with higher rates in at-risk populations [8–12]. Treatment of these pathologic fractures is complex, as these fractures are highly prone to delayed union and nonunion and often require specialized treatment techniques. Given that RT plays an indispensable role in multimodal therapy for STS, a complete understanding of the mechanisms and risk factors for postradiation fracture is important to guide strategies to prevent and treat these fractures. Our objective is to review the mechanisms, risk factors, treatment modalities, and challenges associated with postradiation fractures in STS patients.

## 2. Pathologic Changes in Bone due to Radiation

The effects of radiation on bone can cause osteopenia, osteonecrosis, fracture, and impaired healing [4]. These clinical manifestations result from a combination of several pathologic mechanisms, which include both cellular and related architectural changes in bone, all of which lead to alterations in the mechanical strength of bone [13–16]. These changes appear to be dose-dependent, with cellular changes observed in bone mesenchymal stem cells in animal models with doses as low as 1-2 Gy. At doses higher than 10 Gy, cell survival is decreased, with corresponding changes in architecture, and cell death can be observed at doses of 50 Gy and above [4, 13, 17, 18]. Grossly, radiation-induced bone changes are thought to be location dependent, with demineralization prominent in short tubular and flat bones and irregular cortical thickening occurring in long bones [3].

### 2.1. Cellular Changes

Ionizing radiation therapy causes double-stranded DNA damage in the areas receiving radiation therapy, and these changes can manifest themselves as apoptosis, necrotic death, mitotic death, and radiation-induced senescence [19]. Thus, the effects of ionizing radiation are particularly lethal to tumor cells but have a differential impact on normal tissues, including osteoclasts, osteoblasts, pericytes, osteocytes, and progenitor cells [13, 20, 21]. Specifically, osteoclasts respond in a biphasic manner, with early, transient increases in number followed by later, persistent decreases [14, 15]. The loss of osteoclasts impairs bone homeostasis, leading to poor bone turnover and allowing for the accumulation of microdamage [13–15]. Osteoblasts also show decreased proliferation after exposure to ionizing radiation [22, 23], though the radiosensitivity of osteoblasts does appear to be less than that of osteoclasts [16]. Bone marrow mesenchymal stem cells are also directly affected by radiation, with decreases in osteogenic differentiation and a shift toward adipocyte differentiation [24, 25]. This is detrimental to bone formation and adds to increased marrow adiposity.

### 2.2. Architectural Changes

Intimately related to cellular changes are architectural changes to bone that occur after exposure to ionizing radiation. Decreased vascularity within bone has been noted in several studies [26]. Green et al.'s study also found reduced total new vessel formation at fracture sites, suggesting vascular damage as another potential contributor to RT-induced pathologic changes in bone [23]. Takahashi et al. studied the effects of high-dose RT on Haversian systems in rabbits. Four weeks after a single dose of radiation, dilation of microvasculature and occlusion of Haversian vessels with cell loss were noted, along with abnormal osteoclastic resorption of perivascular bone matrix [13]. This abnormal resorption was not coupled to new bone formation, resulting in increased porosity of the irradiated bone. At 12 weeks after radiation exposure, decreased vascularity became prominent.

Changes in bone morphology have been well studied in animal models. This includes loss of trabecular bone, most pronounced at the metaphysis, and increased cortical thickness [14, 15]. Oest et al. theorize that the persistent late decrease in the osteoclast population leads to unopposed cortical matrix deposition. Without regular bone turnover, which is interrupted by loss of osteoclasts, microdamage accumulates and leads to brittleness and fracture risk [14].

### 2.3. Mechanical Changes

Radiation-related cellular and architectural changes in turn affect the mechanical strength of bone. Sugimoto et al. demonstrated a significant decrease in bending strength 12 weeks after exposure to a single dose (50 Gy) of RT in rabbits [27]. At 24 weeks, bending strength was reduced by half, with a trend toward recovery at 52 weeks. These changes in mechanical strength were synchronous with changes in bone porosity, hematopoietic cells in bone marrow, and endosteal new bone formation. Similarly, femur strength tested by 3-point bending was decreased in mice exposed to 5 Gy x 4 fractions [15]. In this study, tissue mineral density and material properties were felt to be key determinants of decreased strength after RT. When fractures do occur, final strength after repair is also decreased. Pelker et al. studied fracture healing after radiation in a rat model and found delayed healing and decreased final repair strength after healing compared to control groups [28]. The mechanical changes in bone have not been evaluated under fatigue testing conditions but are thought to diminish the fatigue strength of bone substantially.

## 3. Postradiation Fracture

We define a postradiation fracture as one that occurs within a prior radiation field and is not brought on by significant trauma. The literature cites fracture rates between 2 and 22% in STS patients after combined treatment with surgery and RT, though most recent studies report rates closer to 6% [8–12]. These fractures tend to occur years after initial treatment, with the median time to fracture ranging from 24 months [11] to 41 months [9]. While fractures can occur at any irradiated site, the femur is the most frequent and best-studied fracture site.

## 4. Risk Factors for Fracture

Several studies have investigated risk factors for postradiation fracture (Table 1). Commonly identified risk factors can be broadly grouped into either patient factors, which include sex, age, and anatomic location, or treatment-related factors, which include radiation dose, radiation modality, periosteal stripping at the time of surgery, use of chemotherapy, and timing of RT. It should be noted that there is no single risk factor that is universally agreed upon in the literature. This may be due to the low incidence of post-RT fractures. In this light, patients and risk factors must be assessed broadly, rather than strictly based on any single variable.

### 4.1. Patient Factors

#### 4.1.1. Sex

Women have a lower bone mineral density (BMD) and higher fracture risk than men. Differences in fracture risk are due to not only BMD, but also bone size, geometry, and strength [36, 37]. The addition of radiation-induced bone changes to these sex differences is likely additive [12]. Indeed, several studies have demonstrated sex to be a significant risk factor for postradiation fracture in STS patients, both by univariate and multivariate analysis [9–12]. For example, Holt et al. showed female sex to be independently significant in a Cox proportional-hazards model (*P*=0.04). However, not all studies have found a significant association [8, 30].

#### 4.1.2. Age

There are mixed findings regarding age as a risk factor for postradiation fracture in STS patients. This may be partially due to the fact that age is commonly assessed as both a continuous and a dichotomous variable in analyses. Holt et al. demonstrated age (continuous variable) to be an independent risk factor for postradiation fracture (*P*=0.001) [9]. Gortzak et al. and Rimner et al. demonstrated age (as a continuous and dichotomous variable, respectively) to be a significant risk factor for fracture on both univariate and multivariate analyse, whereas Helmstedter et al. found no association between age and fracture risk [10, 12, 30]. Further, Bishop et al. showed age to be significantly associated with fracture when treated as a continuous variable in competing risk analysis, but a significant association was not found using other forms of analysis [8]. Similarly, Lin et al. demonstrated age >50 years as an independent risk for fracture on univariate but not multivariate analysis [11]. One confounder to identifying increasing age as a risk factor for postradiation fracture is that this trend parallels that of the general population, where increased age is a well-known risk factor for fracture [38, 39].

#### 4.1.3. Location

Specific anatomic sites have been associated with a higher risk for postradiation fractures. Most studies on the topic have focused on the lower extremity, specifically the thigh, given that most postradiation fractures occur in this location. In a study of postradiation fractures in the lower extremity, the femur was involved in 71% of fractures, with additional sites of fracture including the tibia, fibula, patella, and metatarsal [32]. Predominant femur involvement parallels the incidence of STS, which is most common at the thigh [40]. This fact combined with the weight-bearing nature of the lower extremities is a likely reason that femora are more prone to fracture in the postradiation setting. Upper extremity postradiation fractures do occur, albeit at a much lower rate. In one study of fractures after combined surgery and radiation for STS that included upper extremity sites, the upper extremity sustained a fracture in 4/20 fracture events, whereas the lower extremity was involved in 16/20 events. The most commonly involved upper extremity bones included the ulna followed by the humerus [30]. The lower frequency of upper extremity postradiation fractures may not be surprising when the lower incidence of soft tissue sarcoma in the upper extremity compared to the lower extremity is considered.

In studies that focus on the femur alone, anterior compartment tumors of the thigh have been variably found to confer a higher risk of fracture, with some studies demonstrating a significantly higher risk [12, 30, 41]; others found a trend toward significance [11, 41]. Still, others found no significant association [9, 10]. Helmstedter et al. theorized that lesions and treatment to the anterior compartment location place stress on the tension side of the femur, increasing the risk of fracture in the postradiation setting [30]. Gait analysis also shows that the quadriceps are active during heel strike, muffling acute load transfer to the femur. We theorize that the absence of the resected anterior muscle changes the rate of loading and may affect the mechanical function of the femur. Regarding location within the femur, fracture at the diaphysis is more common than at the metaphysis (85% versus 15%) [33]. The reasons are not clear. When studying location within the prior radiation field, Holt et al. found that 78% of postradiation fractures occurred in the high-dose central part of the radiation field [9].

Bishop et al. suggested that the amount of femoral circumference exposed to radiation is a significant risk factor for postradiation fracture [8]. This study found that patients treated with RT to the entire circumference of the femur were at significantly higher risk of fracture compared to those who had a portion of the femoral circumference spared [8]. This finding is consistent with Folkert et al. who hypothesized that the association between lower fracture rate and the use of intensity-modulated radiation therapy (IMRT) is due to sparing of a portion of the circumference of the femur [35].

### 4.2. Treatment-Related Factors

#### 4.2.1. Radiation Dose

Although several studies have attempted to define a definitive dose associated with radiation-associated fractures, no clear threshold has been identified. In a retrospective review of the use of limb-sparing surgery and external beam radiation therapy (EBRT) in 364 patients with lower extremity STS, Holt et al. showed a significant increase in the frequency of radiation-associated fractures with high-dose radiation, defined as >60 Gy, compared to low-dose radiation, defined as 50 Gy (7% vs. 0.6% at 5 years, respectively, *P*=0.007) [9]. Pak et al. examined 131 patients with lower extremity STS treated with limb-sparing surgery and EBRT and found that, for all fracture cases with 3D treatment planning data available (*n* = 4 of 5 total fractures), fractures occurred in patients who received mean radiation doses greater than 40 Gy [34]. Similarly, a review of patients with lower extremity STS treated with limb-sparing surgery and EBRT found a mean dose of 37 Gy to bone in nonfracture patients (*n* = 53) compared to 45 Gy in fracture patients (*n* = 21) [32]. This study also found the mean dose to the actual fracture site to be even higher at 59 Gy. This finding highlights the important relationship between dose, volume, and anatomic site targeted for radiation. Thus, it is challenging to evaluate the true risk of fracture as a function of dose. Therefore, while many studies suggest that higher radiation dose is associated with increased fracture risk, other studies have not demonstrated dose to be a risk factor [11, 30].

#### 4.2.2. Radiation Modality

EBRT is the best-studied radiation modality in terms of risk of postradiation fracture, although the fracture rate varies across studies. Additionally, can be delivered using a variety of techniques, which variably allow increased sparing of normal tissues. Studies in STS have reported fracture rates of 6.0%, 7.7%, and 22.0%, respectively, in patients receiving combined EBRT and surgery [12].

With increased sophistication in diagnostic imaging and more advanced planning techniques, radiation oncologists are IMRT with image guidance in order to spare nearby organs at risk, including bone [42, 43]. Studies have suggested a lower risk of fracture in patients treated with IMRT compared to conventional 2-dimensional (2D) or 3-dimensional (3D) conformal radiation treatment [35]. For example, Folkert et al. observed a fracture rate of 6.5% after IMRT in a consecutive series of 92 STS patients, a rate that was significantly less than the expected fracture rate in patients treated with conventional 2D or 3D conformal treatment (25.6%) as calculated by the Princess Margaret Hospital nomogram [35]. The relative decreased fracture risk with IMRT may be due to either the reduced volume or circumference of bone receiving high-dose radiation. This hypothesis is supported by data from Bishop et al. who found no fractures when a portion of the femoral circumference was spared and a 7% fracture rate when the entire circumference of the femur was treated (*P* < 0.001) in a cohort of 596 patients after treatment with combined surgery and RT [8].

Traditionally, external beam radiation has been delivered with linear accelerators generating photons in order to treat and target cancer cells, with advancements in imaging, planning, and immobilization allowing more conformal (or highly shaped) RT. Proton therapy is a type of external beam radiation that takes advantage of the characteristic Bragg-peak curve [44], where radiation can be delivered to a certain depth in tissue, but does not exit through the tissue, allowing better sparing of normal tissues distal to the target. Proton therapy can be used either using simple planning techniques (3D) or with more conformal planning approaches (such as pencil-beam scanning) in order to generate plans where the normal tissue beyond the distal edge of the tumor can be spared with low-dose radiation therapy. Because of the data suggesting that sparing the circumference of the bone has some correlation with fracture risk, it is possible that proton therapy can spare the distal half of the bone from receiving even low doses of radiation, but it is entirely dependent on the tumor location and patient anatomy. Currently, a single-arm phase 2 clinical trial is underway to evaluate late radiation morbidity (ClinicalTrials.gov identifier: NCT01819831).

Brachytherapy, a technique that uses radioactive sources that act over a short distance, often involves intracavitary applicators, surface catheters, or implanted catheters placed directly into or around high-risk areas. As a result, the dose distribution of the radiation is often more conformal, or highly targeted, with less normal tissue receiving high-doses of radiation therapy. Depending on the placement of the applicators and the dose distribution, this may reduce the total volume and the circumference of bone radiated, thereby potentially reducing the impact of radiation on bone and risk for subsequent fracture [45]. In a review of the use of adjuvant brachytherapy for patients with high-grade STS of the extremities, Alektiar et al. reported a 3% five-year actuarial rate of fracture [46]. Lin et al. showed a significant difference between the two modalities, with a 21% fracture risk for EBRT versus a 5% risk for brachytherapy at five years (*P*=0.043) in a series of 205 consecutive patients [11]. In a study by Rimner et al., adjuvant EBRT was associated with a significantly higher five-year probability of bone fracture compared to brachytherapy in a series of 225 patients (13.4 vs. 3.7%, respectively, *P*=0.01), although this finding did not remain significant on multivariate analysis [10]. Finally, Thomas et al. reported only one fracture (2%) after brachytherapy in a cohort of 48 STS patients where brachytherapy was performed either as part of initial treatment (*n* = 27) or in patients presenting with relapse (*n* = 21) [47]. While brachytherapy is not currently in favor at many institutions, likely due to time, cost, logistics, and patient comfort, these studies suggest a reduced fracture rate as compared to EBRT.

### 4.3. Periosteal Stripping

The periosteum is an excellent source of progenitor cells, which aid in bone healing and regeneration. Studies have found periosteum-derived cells to be at least comparable to, and sometimes superior to, bone marrow-derived multipotent mesenchymal stromal cells [48]. Therefore, it is not surprising that periosteal stripping at the time of surgery is a frequently identified factor associated with increased risk for radiation-associated fractures. Early on, Brant et al. observed that the periosteum had been stripped in 3 of 4 fracture cases in their series [29]. Using a multivariate Cox regression model, Lin et al. identified periosteal stripping as the only independent factor predictive of fracture [11]. Helmstedter et al. classified the extent of periosteal stripping in 163 patients with thigh tumors treated with surgery and radiation for STS as none, minimal (<10 cm), moderate (10–20 cm), or extensive (>20 cm). The authors found that patients who had moderate or extensive stripping at the time of surgery were 18 times more likely to have fractures than patients with no or minimal stripping [30]. More recently, Bishop et al. showed that bone exposure and periosteal stripping during surgical resection were significantly associated with fracture after radiation [8]. Despite these studies suggesting that periosteal stripping increases the risk of postradiation fracture, similar to the other risk factors discussed herein, there is a lack of consensus. Multivariate analysis did not show an association between periosteal stripping and fracture in the Holt et al.'s study [9], and Gortzak et al. [12] did not find periosteal stripping to be significantly associated with fracture in a multivariate logistic regression analysis [30]. Rather than indicating that periosteal stripping does not matter, these discordant findings may highlight the difficulty in accurately quantifying periosteal stripping and powering studies on the subject.

### 4.4. Chemotherapy

Chemotherapy is known to affect bone physiology. For example, doxorubicin, a commonly used chemotherapeutic agent in STS, was shown to have toxic effects on osteoblast function that lead to profoundly diminished bone formation rates in a rat model [49]. Studies have found the administration of either neoadjuvant or adjuvant chemotherapy to be a significant risk factor for postradiation fractures. Bishop et al. showed significantly increased rates of fracture in patients receiving either neoadjuvant or adjuvant chemotherapy on both time to fracture analysis (*P*=0.02) and univariate competing risk analysis (*P*=0.03), and Lin et al. demonstrated administration of chemotherapy to have significant prognostic value for fracture (*P*=0.02) [8, 11]. Similar to other risk factors, however, this finding has not been universal, with other studies finding no significant association between chemotherapy use and postradiation fracture [34, 35]. As chemotherapy use in STS is evolving, continued exploration of its role in postradiation fracture is needed.

### 4.5. Neoadjuvant versus Adjuvant RT

The evaluation of radiation administration in either the neoadjuvant or adjuvant setting is intimately tied to the evaluation of radiation dose and field. Lower doses with more narrow treatment fields are commonly administered preoperatively, whereas higher doses and wider fields are necessary after surgical intervention. While it might seem intuitive that higher doses to larger areas of bone in the adjuvant setting might lead to increased fracture risk, few studies have specifically studied the effect of the timing of RT on postradiation fractures. To date, no clear difference has been established [30, 32, 34].

## 5. Treatment of Postradiation Fractures

### 5.1. Challenges

Early literature on postradiation fractures suggested that irradiated bone healed differently from nonirradiated bone. There was evidence, albeit limited, that the average time to union was increased and nonunions were more common for fractures in previously irradiated bones [3]. More recent studies have confirmed these early observations, finding a nonunion rate of approximately 45% in STS patients with postradiation fractures [11, 30, 32]. These effects have been attributed largely to radiation-induced changes. In an animal model of postradiation fracture, minimal osteoblast proliferation and reduced total new vessel formation were observed at fracture sites, with reduced new bone formation [23]. The poor healing potential of postradiation fractures is likely due to damage to regenerative cells and microvasculature in the region of the fracture. Given this unique pathology, these fractures require specialized clinical management.

Infection is another significant challenge when treating postradiation fractures and is related to a compromised soft tissue envelope, poor vascularity, extensive fibrosis, necrotic bone, and frequent need for multiple surgeries [50, 51]. Deep infection rates up to 20% after surgery for postradiation fractures have been reported in some series [30, 50]. Infection prevention is therefore imperative, and minimizing the number of surgeries is an important strategy to decrease the risk of infection [51]. A high level of suspicion for wound healing difficulty and infection problems should be maintained. Procedures should be planned and performed with great care [51]. The use of soft tissue coverage, including vascularized free-tissue transfer, for wound closure in irradiated tissue has been associated with reduced complications, decreased need for secondary procedures, and greater limb salvage rates [52, 53].

### 5.2. Fracture Management

Kim et al. studied 37 STS patients with 39 postradiation fractures and noted a nonunion rate of 63% in patients treated with open reduction and internal fixation (ORIF) [33]. Diaphyseal location and displacement of the fractures were important predictors of nonunion in this population. In contrast to patients treated with ORIF, patients treated with endoprosthetic reconstruction had no major complications, including nonunion, and did not require revision procedures in the follow-up period (mean follow-up is 12.2 years for the whole cohort; follow-up time after endoprosthetic reconstruction is not specified). Based on these data, the authors advocated for an aggressive approach when treating postradiation fractures to minimize complications [33].

Bone morphogenetic protein 7 (BMP-7), part of the transforming growth factor-beta superfamily of proteins, is known to be involved in fracture healing and has been shown to improve rates of fracture union [54–56]. Given the role of BMP-7 in nonunion, Nicholls et al. investigated the role of local administration of BMP-7 in a rat model of post-RT fracture but failed to demonstrate a clinical benefit to healing. Therefore, the authors suggested that radiation and surgical periosteal stripping compromised healing mechanisms beyond what can be compensated for by BMP-7 alone [57].

Given the difficulty in achieving union after ORIF, vascularized free fibula grafting has been investigated as a potential adjunct for healing. In a study of 18 patients with postradiation fractures, Duffy et al. found 83% of fractures united primarily with the use of vascularized free fibula to bridge the fracture site at an average of 9.4 months postoperatively. Of those that did not unite primarily, one patient achieved union after a secondary bone grafting procedure, while two did not unite [58]. There was a 22% rate of deep infection in this study, and all patients with deep infections required subsequent surgery. In contrast, Kim et al. did not observe the same success rate with vascularized free fibula grafting, although the sample size was small. In four patients who received a graft after prior failed fixation attempts, two healed and two had major problems with infection and wound healing [33]. While the use of a vascularized free fibula grafting appears to increase union rates in some series, complications common to all postradiated tissues present a surgical challenge. For cases of recalcitrant nonunion, amputation may be required and has been used as a treatment strategy in extreme cases [11, 31].

### 5.3. Case Example

**Figure 1:**
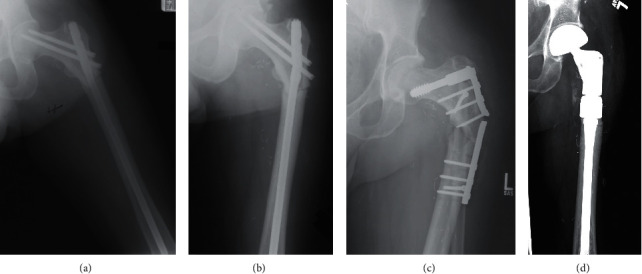
Postradiation fracture in a 55-year-old male who had excision of a left thigh liposarcoma and postop radiation (64 Gy). The fracture occurred 4 years after treatment. (a) He was initially treated with an intramedullary device, (b) which eventually broke due to nonunion at the fracture. The patient went on to revision with a sliding hip screw and side plate with a free fibula transfer. (c) This also went on to failure at the site of the postradiation fracture. (d) He was then revised to a proximal femoral replacement, which remains stable.

Figure 1 highlights several of these treatment challenges. The patient presented provided consent, allowing his case to be presented in this report. The patient, a 55-year-old male, initially underwent surgical resection and postoperative EBRT with 64 Gy for myxoid liposarcoma of the posterior thigh. He later sustained a postradiation subtrochanteric femur fracture four years after treatment. Initially, he was treated with an intramedullary device. The fracture went on to nonunion, and the device failed. The patient was then revised to a sliding hip screw and side plate with a concomitant ipsilateral-free fibula transfer to address the nonunion. This also went on to failure, with persistent nonunion and fracture of the plate. Then, the patient was revised to a proximal femur endoprosthetic replacement, which has been stable for more than 15 years. At the time of this revision, the bone was noted to be brittle and avascular, without evidence of callus formation at the fracture site. At the most recent follow-up, the patient was ambulating with a cane and without signs of hardware complication. He remains disease-free.

## 6. Fracture Prevention

### 6.1. Prophylactic Fixation

Given the challenges that postradiation fractures pose to patients and surgeons, prevention is of great interest. Many advocate for prophylactic intramedullary nailing of the femur in patients undergoing STS resection, especially when periosteal stripping is involved [11, 30, 34]. Pak et al. found a substantially lower rate of fracture in patients treated with RT and surgery involving periosteal excision and prophylactic intramedullary nailing of the femur as compared to similar at-risk patients from other series who had undergone periosteal stripping (8% vs. 30%) [34]. The prophylactic procedure was associated with a minimal additional operative time, a relatively short recovery time, and a low risk of complications, all of which support the addition of prophylactic nailing to the surgical plan for these high-risk patients [34]. However, a common concern with the use of prophylactic intramedullary nails is that they create a metal artifact that could obscure future imaging studies and potentially interfere with evaluation for local recurrence [11]. At our institution, we frequently use carbon-fiber implants in these cases to minimize metal artifact [59].

Another concern with prophylactic fixation is overtreatment. Although postradiation fracture is associated with high morbidity, relatively few patients exposed to RT will experience a fracture. Thus, there is a need for methods to identify patients who would most benefit from prophylactic fixation. Lin et al. developed an algorithm for determining when to perform prophylactic fixation specific for the femur that considers periosteal stripping, sex, chemotherapy, and compartment location [11]. The authors found that eight of nine fractures would have been prevented with the use of the algorithm in their series of 17 patients with prophylactic nailing. Gortzak et al. included periosteal stripping, sex, compartment location, radiation dose, tumor size, and age at index surgery in a multiple regression model to predict radiation-associated fracture risk. The model was 91% sensitive and 81% specific, with an area under the receiver operating characteristic curve of 0.9; a nomogram was developed based on the predictive model [12]. Neither of these models has been tested in outside patient populations; validation and prospective study will be necessary to develop clinical tools to aid in risk stratification and allow for the identification of patients who would benefit most from prophylactic fixation.

### 6.2. Future Directions for Fracture Prevention: Medical Therapies

Several studies suggest a role for medical therapies to prevent and treat damage to bone caused by RT. Most are still experimental, but studies from animal models are promising. These therapies include the use of antioxidants, parathyroid hormone (PTH), and antiresorptive medications including bisphosphonates and denosumab [18].

RT leads to the formation of free radicals that damage the bone marrow microenvironment. Antioxidants such as ascorbic acid (vitamin C), *α*-lipoic acid, and misoprostol have been shown in animal models to lessen the damage to bone after RT [60–62]. Vitamin E, or tocopherol, is another antioxidant shown to act synergistically with pentoxifylline, a phosphodiesterase inhibitor that acts to vasodilate and inhibit inflammatory reactions, to reduce chronic damage from RT [63, 64]. A case series by Bohn et al. showed successful treatment of mandible osteoradionecrosis using this combination of medications [63]. Another study by Robard et al. similarly showed improvement in osteoradionecrosis of the mandible using pentoxifylline and tocopherol with the addition of clodronate [65]. While these series differ from common orthopedic manifestations of fracture, their findings are promising and suggest a role for investigation in extremity fractures.

Amifostine is a synthetic antioxidant shown to lessen tissue damage by binding free radicals and promoting tissue repair [66]. It has been shown in both animal models to protect bone quality and healing, though most of this work focuses on the mandible rather than long bones [67–69]. Additionally, amifostine has been shown to be radioprotective to the physis in combination with misoprostol, selenium, and pentoxifylline [70].

Controversy exists regarding timing of treatment with antioxidant therapies. Given that the antitumor effects of radiation are thought to occur through the creation of free radicals, the administration of antioxidants during treatment raises theoretical concern for decreasing the efficacy of treatment. Therefore, many oncologists avoid administration during active treatment, reserving use until therapy is completed [71]. However, studies have not shown detrimental effects with concomitant administration of radiation with antioxidants [71–73]. There appears to be less concern about use of amifostine during radiation treatment, with several studies showing that concurrent use did not affect treatment outcome [71, 74–76]. Further arguing against detrimental effects from concomitant administration, a study showed that razoxane, another synthetic antioxidant, increased response rate when administered with RT for STS, compared to treatment with RT alone [77].

PTH is involved in calcium and phosphorous homeostasis, whereby intermittent administration is known to lead to bone formation. Its use has been shown to attenuate radiation-induced damage by osteoblasts by promoting repair of DNA double-stranded breaks in a preclinical cellular model [78]. In another study, PTH treatment prevented deterioration of bone trabeculae caused by radiation, prevented the loss of mechanical competence of bone, rescued both osteoblasts and precursors from radiation-induced damage, and abrogated radiation-induced cell death in the marrow [79]. In another biomechanical study, a rat model of irradiated mandibular distraction osteogenesis demonstrated that PTH treatment was successful in improving several bone healing parameters, including ultimate load, failure load, and yield [80].

Bisphosphonates have been shown to prevent trabecular bone loss after treatment with RT in mouse models. However, a mechanical advantage has not yet been proven, suggesting that further investigation is necessary [81, 82]. In a prospective, randomized clinical trial in 2014, investigators evaluated the effects of zoledronate on radiation-induced degradation of bone collagen by measuring urinary excretion of bone metabolites and found that zoledronate was able to prevent early radiation-induced collagen degradation. They concluded that radiation-induced bone loss is a consequence of osteoclastic bone resorption, which the bisphosphonate appeared to have the ability to counteract [83]. Conversely, the Cochrane Database reviewed two clinical trials examining the effect of bisphosphonate administration in men undergoing pelvis radiation for prostate cancer; insufficient evidence was found to show that bisphosphonate administration prevented radiation-induced bone complications [84]. Finally, denosumab has been theorized to have radioprotective effects as well, given its function to inhibit osteoclasts [18].

### 6.3. Multidisciplinary Discussion

Patients undergoing both surgical resection and RT for treatment of STS represent a complex population. While the overall incidence of postradiation fracture is low, for the patients affected, it is a challenging problem with significant effects on quality of life, making fracture prevention a critical aspect of care that deserves deliberate discussion. Although the previously noted predictive models provide a framework to consider prophylactic fixation of bone, we recommend that all patients undergoing combined modality treatment of STS involving surgery and RT have their management discussed by a multidisciplinary team with experience managing STS. This critical step helps inform the radiation oncologists of the surgical approach, which helps them to better spare normal tissue in the surgical bed and target areas at risk. Multidisciplinary discussion also aids in identifying patients at high risk for postradiation fracture, as well as other surgical complications. Tumor control must be paramount, so such a multidisciplinary discussion allows providers to discuss opportunities to minimize risk while maximizing local control, and to consider prophylactic treatment when multiple risk factors are noted. Input from medical oncologists is recommended in cases where patients have or will receive chemotherapy. Additionally, we believe that discussion of postradiation fracture risk with patients should be a dedicated portion of the informed consent process.

## 7. Conclusions

Postradiation fractures are a significant problem in orthopedic oncology. While overall rates are low, these injuries pose a significant treatment challenge—radiation causes vasculature damage and impairs osteoclast and osteoblast function, which impairs healing. Common risk factors for postradiation fractures include high radiation dose, female sex, periosteal stripping, older age, femur location, and chemotherapy administration. Despite the fracture risk, radiation is a critical component of treatment for STS; due to oncologic risk, dose reduction or radiation avoidance should not be advocated. Advancements in radiation planning and delivery, including IMRT and proton therapy, may allow sparing of normal bone, depending on the anatomic considerations and target volume. Currently, there is a lack of data on how these techniques directly translate into mitigation of the risk for post-RT fractures, making continued study and multidisciplinary planning critical to improve future care. In cases where portions of bone cannot be spared or when risk is sufficiently high, prophylactic stabilization should be considered. Carbon-fiber-based implants can be used in these cases to minimize metal artifacts that could obscure future surveillance studies. Radioprotective medications may provide additional protection and treatment options but require further clinical studies to fully elucidate their role. Continued assessment of risk factors and identification of novel approaches to preventing and treating postradiation fractures are necessary to improve upon current techniques.

## Figures and Tables

**Table 1:** Studies of risk factors for postradiation fractures in patients with STS treated with surgery and RT.

Author	Year	No. of patients	No. of fractures	STS location	Significant variables identified	Reference
Brant et al.	1990	52	4	Long bone	Periosteal stripping, age, female gender; descriptive only	[29]
Lin et al.	1998	205	5	Thigh	Univariate: compartment, periosteal stripping, female gender, chemotherapy, age, RT type Multivariate: periosteal stripping Secondary multivariate model: female gender, chemotherapy	[11]
Helmstedter et al.	2001	285 (thigh subset: 163)	20 (thigh subset: 12)	Extremity	Logistic regression (thigh subset): periosteal stripping, compartment, marginal/intralesional resection	[30]
Holt et al.	2006	354 (thigh subset: 239)	27 (thigh subset: 24)	Lower extremity	Univariate: periosteal stripping, compartment, age, radiation dose Multivariate: age, radiation dose Univariate (thigh subset): female gender, age Multivariate (thigh subset): female gender, age, radiation dose, radiation timing	[9]
Livi et al.	2005	214	7	Extremity	None	[31]
Dickie et al.	2009	691	31^a^	Lower extremity	Radiation dose (mean, maximum, proportional volume irradiated)	[32]
Gortzak et al.	2010	101	22	Thigh	Univariate: age, tumor size, periosteal stripping, compartment Multivariate: age, tumor size, gender	[12]
Kim et al.	2010	1045	39	Thigh	Diaphyseal location; descriptive only	[33]
Pak et al.	2012	131	5	Proximal lower extremity	Radiation dose^b^	[34]
Bishop et al.	2016	596	12	Proximal lower extremity	Univariate competing risk; bone exposure, periosteal stripping, chemotherapy, age Time to fracture analysis: bone exposure, periosteal stripping, chemotherapy	[8]
Folkert et al.	2019	92	6	Thigh and groin	Univariate: age (<60), compartment, periosteal stripping Multivariate: age (<60), periosteal stripping IMRT rate < expected rate for EBRT	[35]

^a^24 fractures (in 21 patients) could be analyzed, ^b^Dosimetric analysis limited to 4 fractures. STS: soft tissue sarcoma; RT: radiation therapy; No.: number; IMRT: intensity-modulated radiation therapy; EBRT: external beam radiation therapy.
